# Association between dietary protein intake, diet quality and diversity, and obesity among women of reproductive age in Kersa, Ethiopia

**DOI:** 10.3389/fpubh.2023.1258515

**Published:** 2023-11-14

**Authors:** Aklilu Abrham Roba, Nega Assefa, Kedir Teji Roba, Yadeta Dessie, Elena Hamler, Wafaie Fawzi

**Affiliations:** ^1^College of Health and Medical Sciences, Haramaya University, Dire Dawa, Ethiopia; ^2^Faculty of Health Science, Erciyes University, Kayseri, Türkiye; ^3^Harvard T.H. Chan School of Public Health, Boston, MA, United States

**Keywords:** protein intake, obesity, overweight, MDD-W, global dietary quality score, reproductive age women, Ethiopia

## Abstract

**Introduction:**

In Ethiopia, there is limited evidence on the effect of dietary protein intake on women’s body mass index. Therefore, this study investigated the association between dietary protein intake, diet quality, and overweight and obesity.

**Methods:**

A cross-sectional study was conducted among 897 women of reproductive age. Food frequency questionnaires were used to assess 7-day dietary intake. It was converted into protein and other macro-nutrient intakes, Minimum Dietary Diversity for Women, and Global Dietary Quality Score. Body Mass Index (BMI) of overweight & obese women were defined as ≥25 kg/m^2^. An adjusted odds ratio with a 95% confidence interval (in a multivariate logistic regression model) was used to determine the strength of the association between BMI and dietary protein intake, adjusting for potential confounders.

**Results:**

The median dietary protein intake was 41.3 (32.9, 52.6) grams/day or 0.8 (0.6, 1.0) grams/kilogram of body weight/day. The prevalence of overweight and obesity was 7.5% (*n* = 67). Only 220 (24.5%) women could meet the recommended minimum dietary diversity of five or more food groups out of 10 per day. Furthermore, only 255 (28.4%) women were found to have a low risk for nutrient adequacy. Interestingly, women who consumed moderate dietary protein had a significantly lower likelihood of being overweight or obese, with AOR of 0.21 (95% CI 0.10–0.48). Similarly, those who consumed a high amount of protein had even lower odds, with AOR of 0.03 (95% CI 0.01–0.14), compared to those who consumed a low amount of dietary protein. Age of 40–49 years (AOR = 3.33, 95% CI 1.24–8.95) compared to 18–29 years, non-farmers (AOR = 3.21, 95% CI 1.55–6.62), higher consumption of food from unhealthy groups (AOR = 1.30, 95% CI 1.05–1.61), and high fat intake (AOR = 1.06, 95% CI 1.04–1.09) were associated with overweight and obesity.

**Conclusions and recommendations:**

The study indicated an inverse relationship between BMI and dietary protein intake. It also revealed that women who consumed foods from unhealthy or unhealthy when consumed in excessive amounts were more likely to be overweight or obese. Increasing dietary protein consumption can help reproductive-age women reduce the odds of obesity and overweight. Furthermore, community-based educational programs, policy changes, and healthcare services can support this effort.

## Introduction

The definition of diet quality varies depending on dietary habits, cultural background, the availability of local foods, and individual requirements (such as age, gender, and level of physical activity). However, it can be stated as the adequacy of essential nutrients and energy in supporting bodily functions, promoting well-being, facilitating physical activity, preventing infections, and reducing the risk of diet-related noncommunicable diseases (1). A diet consisting of lower-quality foods, such as highly processed snacks, sugary drinks, white grains, refined sugar, fried foods, foods high in unhealthy fats, and high-glycemic foods like potatoes can lead to weight gain, obesity and other chronic conditions such as diabetes, cancer, heart diseases, and stroke (2–4). Conversely, high-quality foods consist of unrefined, minimally processed options like vegetables, fruits, whole grains, nuts, pulses, healthy vegetable and seed oils, healthy fats, and healthy sources of plant and animal proteins (3, 5). Consuming high-quality foods in appropriate portions helps in maintaining good health and attaining an ideal body weight without obesity, while also reducing inflammation within the body (3, 6).

In the past, the absence of a single, validated index to assess diet quality in low- and middle-income countries has led to the utilization of proxy measures like dietary diversity scales. However, in recent years, standardized tools like the Global Dietary Quality Score (GDQS), has emerged to measure diet quality (7, 8). A lower score has been linked to an increased risk of obesity and its related complications, whereas higher scores have been associated with more favorable anthropometric measurements, including lower body mass index, waist-to-height ratio, and waist circumference (9, 10).

Globally, the prevalence of overweight and obesity has increased in the last few decades, including in low- and middle-income countries (11). As the global prevalence tripled between 1975 and 2016, the same pattern continued in many African countries, including Ethiopia (12, 13). It caused 2.4 million deaths and 70.7 million disability-adjusted life years among females in 2017 in the world (14). Overweight and obese women are at risk of chronic diseases, untimely death, mental disorders, postpartum bleeding, and fetal macrosomia (15, 16).

The fundamental cause of overweight and obesity is an energy imbalance between calories consumed and expended (12). It is also well-established that unhealthy eating pattern leads to weight gain (17). Furthermore, overweight and obesity were associated with advancing age, urban residence, female gender, better educational status, and being in the affluent quintile (18, 19). Recent evidence indicates an inverse association between healthy diets, overweight, and obesity (20).

The role of dietary protein in the development of obesity is controversial. Some studies indicated that excess protein intake beyond recommended daily requirements may increase fat-free mass and adiposity, with a high potential to increase BMI (18). On the other hand, high protein and low carbohydrates resulted in significant weight loss among obese adults in interventional studies (21, 22).

In recent years, plant-based proteins have been considered a healthy option to meet the body’s protein demands (23). Plant-based proteins are a significant source of dietary protein, calories, and minerals in many low- and middle-income settings. Though people who consume more plant-based staple foods were challenged by low protein quality (24) and had lower muscle mass index (25), it has also had positive outcomes. For instance, people who obtain protein from plant source has a lower risk of hypertension, cardiovascular disease, metabolic syndrome, and type-2 diabetes (23, 25).

Animal-source proteins (ASP) are energy-dense and contain readily digestible protein along with micronutrients (minerals including iron, zinc, calcium, and vitamins such as vitamins B-12, vitamin A, and riboflavin) that are essential to meet the body’s requirements (26). However, evidence indicates that excess consumption of ASP is associated with hypertension, cardiovascular diseases, type-2 diabetes, and metabolic syndrome (23, 25, 27). On the other hand, a diet rich in plant-based proteins but low in animal-source protein is essential to maintain a healthy condition (28).

Understanding the usual protein intake and other macronutrients among women of reproductive age is essential for evaluating their nutritional status (29). In Ethiopia, there is limited evidence on the effect of dietary protein intake on women’s body mass index. Therefore, this study investigated the association between dietary protein intake, diet quality, and overweight and obesity among women in Kersa district, Ethiopia.

## Methods

### Study area and period

This study was conducted in Kersa Health and Demographic Surveillance Site (HDSS) in the Oromia region of eastern Ethiopia from June to September 2019. The study site mainly consists of rural areas and includes 21 rural kebeles (the smallest administrative unit in Ethiopia), as well as the three small towns of Kersa, Lange, and Weter. The baseline census was conducted in 2007 and updated every 6 months to register demographic and health events. At baseline, 10085 houses, 10,522 households, and 50,830 people were registered. The total fertility rate ranges from 4.0 to 5.3. The details of the study area were published somewhere (30).

### Study design and population

This was a cross-sectional quantitative survey using face-to-face interviews and anthropometric measurements. The study’s eligibility criteria required households to have at least one married woman between the ages of 15 and 49. In cases where multiple women in the household met these criteria, a lottery method was employed to select one woman for the interview. Both breastfeeding and non-lactating mothers were included, but pregnant women (self-reported) were excluded from the study for uniformity of anthropometric values. Based on energy intake, women with less than 500 or greater than 5,000 calories were excluded from the analysis because of its implausibility (31).

### Sample size

As part of a larger study involving 1,200 households with women of reproductive age (15–49 years old) (32), only 897 women were included in this particular study. This is because 303 women were excluded from the analysis based on their pregnancy status (pregnant).

A post-hoc power analysis was conducted using G∗Power 3.1.9.4 software (33) to determine the study’s power. The analysis took into account an alpha level of 0.05, a sample size of 897, and a one-tailed distribution. Based on the odds ratio between women’s body mass index and associated factors such as moderate and high dietary protein intake, non-farmers, total fat and carbohydrate intake, the study found a power of 0.99.

### Data collection and measurements

Research assistants administered a demographic and dietary survey to selected women in the sampled households. The information included the socio-demographic and dietary characteristics of the study population. Individual-based food frequency questionnaires (FFQ) assessed dietary intake, including total, animal, and plant-based proteins, over the previous 7 days and 24 h. The FFQ included all major food groups in 20 clusters (75 food items and drinks), including foods made from grains; pulses; nuts and seeds; milk and milk products; organ meat; meat, fish, and poultry. A 24-h dietary intake was used to calculate MDD-W; otherwise, a 7-day intake was used.

Food matching was made by referencing the Ethiopia Food Composition Table (EFCT) (34). For food items not found in EFCT, mainly fruits and vegetables, the Tanzanian Food Composition Table was referenced (35). Each food’s energy and nutrient values were obtained by cross-referencing the sources cited above. After that, the average daily consumption of food items was calculated by dividing 7-days consumption by 7. Women’s daily dietary protein intake (g) was divided by their body weight (kg) to generate protein intake in units of g/kg body weight/day (31). The recommended dietary allowance (RDA) estimates the minimum daily average dietary intake level that meets the nutrient requirements of nearly 97 to 98% of healthy individuals (36).

The RDA for protein is 0.8 gram/kilogram of body weight/day or 46 grams/day (37). Low protein intake is defined as daily protein consumption below RDA (<0.8 grams/kilogram of body weight/day); moderate intake is from 0.8–1.2 grams/kilogram of body weight/day, whereas high intake is defined as > = 1.2 grams/ kilogram of body weight/day (38).

Minimum Dietary Diversity for Women (MDD-W) was constructed by asking the respondents to recall the foods and beverages consumed with their quantities from each food group in the past 24 h (39). According to MDD-W guidelines, there are ten food groups, namely: 1-grains, roots, and tubers; 2-pulses; 3-nuts and seeds; 4-dairy; 5-meat, poultry, and fish; 6-eggs; 7-dark green leafy vegetables; 8- other vitamin A-rich fruits and vegetables; 9-other vegetables; and 10-other fruits (39). Women who consumed at least five of the ten possible food groups were classified as having minimally adequate dietary diversity (ADD). In contrast, those who consumed less than five were classified as having a low dietary diversity (LDD) (39).

Global Dietary Quality Score (GDQS), the latest tool that measures overall diet quality regarding both nutrient adequacy and diet-related non-communicable disease (NCD) risk, was used. It constitutes 25 food groups, of which 16 are healthy (scored by giving more points for higher intake), seven unhealthy (more points for lower intake), and two food groups are classified as harmful when consumed beyond the acceptable limit (increasing points are given until specific amounts have been consumed, after which no points are given). It is computed by adding all of the 25 food groups ranging from 0 to 49, scoring higher points reflecting a healthier diet. GDQS scores of ≥23 is associated with a low risk of both nutrient adequacy and NCD risk, scores ≥15 and < 23 indicate moderate risk, and scores <15 indicate high risk. Diet-related NCD risks include metabolic syndrome, change in weight and waist circumference, and incident type 2 diabetes (7, 8).

Height and weight were measured twice, and the average was used. With a stadiometer, height was measured to the nearest 0.1 cm with the subject barefoot. Weight was measured to the nearest 0.1 kg with the subject barefoot and in light clothes, using a standard clinical scale. BMI was calculated as weight(kg)/height(m)^2^. Body mass index, or BMI, is defined as weight in kilograms divided by the square of height in meters. The National Institutes of Health (NIH) and the World Health Organization (WHO) have defined overweight as a BMI of 25 to 30 kg/m^2^ and obese as a BMI of 30 kg/m^2^ or higher. A normal BMI range from 18.5 kg/m^2^ to 25 kg/m^2^ (40).

The dependent variable for this study was overweight and obesity, while the primary explanatory variable of interest was dietary protein intake. In addition, this study considered the following as potential confounders: age (years, continuous), women’s lactation status (yes/no), educational attainment (no formal education, attended formal education), if women are married (yes, no), wealth index (poor, middle, wealthy), occupation (farmer, non-farmer). We also examined the relationships of minimum dietary diversity of women (low/adequate), positive and negative global dietary quality score as continuous variables, total fat (continuous), and total carbohydrate (continuous) with overweight/obesity.

### Statistical analysis

The normality of data was determined using Shapiro–Wilk’s test. Both BMI and dietary protein consumption were skewed to the right. Outlier detection was made by visual inspection of histograms and box plots. Therefore, the median values with 25th and 75th percentiles were chosen as the cut-offs to report the findings. A binary logistic regression model was used to determine the association between overweight and obesity and dietary protein consumption taking women with normal BMI as a reference group. An adjusted odds ratio (with 95% CI) was used to determine the strength of the association in models adjusting for intake of fat and carbohydrate, age, wealth index, education, marital, occupation, and lactational status. Statistical significance was determined using a value of *p* <0.05. Analysis was conducted using Stata version 16.0 (41).

## Results

### Socio-demographic characteristics and nutritional status

A total of 897 women (18–49 years) were included in this analysis. The median age of the participants was 30 (27, 35) years. The majority of the participants, 734 (81.8%), were farmers, 849 (94.6%) were Muslims, 876 (97.7%) were married, and 597 (66.6%) were lactating (not pregnant). Based on the wealth index, 33.9, 33.6, and 32.5% of the women were classified into poor, medium, and rich wealth quartiles, respectively. The prevalence of overweight and obesity among women of reproductive age in Kersa was 7.5% (67/897) (Table 1).

**Table 1:** Socio-economic status of reproductive age women in Kersa, 2019.

S/No	Variable		Number	Percent
1.	Education status	No formal education	479	53.4
		Formal education	418	46.6
2.	Occupation	Farmer	734	81.8
		Non-farmer	163	18.2
3.	Lactation status	Lactating (Non-pregnant)	597	66.6
		Non-lactating (non-pregnant)	300	33.4
4.	Religion	Muslim	849	94.6
		Non-Muslim	48	5.4
5.	Marital Status	Married or lives with partner	876	97.7
		Divorce, Widow and Separated	21	2.3
6.	Wealth index	Poor	304	33.9
		Middle	301	33.6
		Rich	292	32.5
7.	Age (years)	18–25	201	22.4
		26–35	522	58.2
		36–49	174	19.4
8.	Residence	Semi-urban	142	15.8
		Rural	755	84.2
9.	Body Mass Index	Normal	675	75.2
		Overweight and obese	67	7.5
		Underweight	155	17.3
10.	Protein intake based on RDA	Low	446	49.7
		Moderate	317	35.3
		High	134	15.0
11.	Minimum dietary diversity	Low diet diversity	676	75.5
		Adequate diet diversity	220	24.5
12.	Energy consumed	Less or equal to 1700 kcal /day	695	77.5
		Higher than 1700 kcal/day	202	22.5
13.	Global dietary quality score	Low risk	255	28.4
		Moderate risk	630	70.2
		High risk	12	1.3

### Minimum dietary diversity for women (MDD-W)

**Figure 1:**
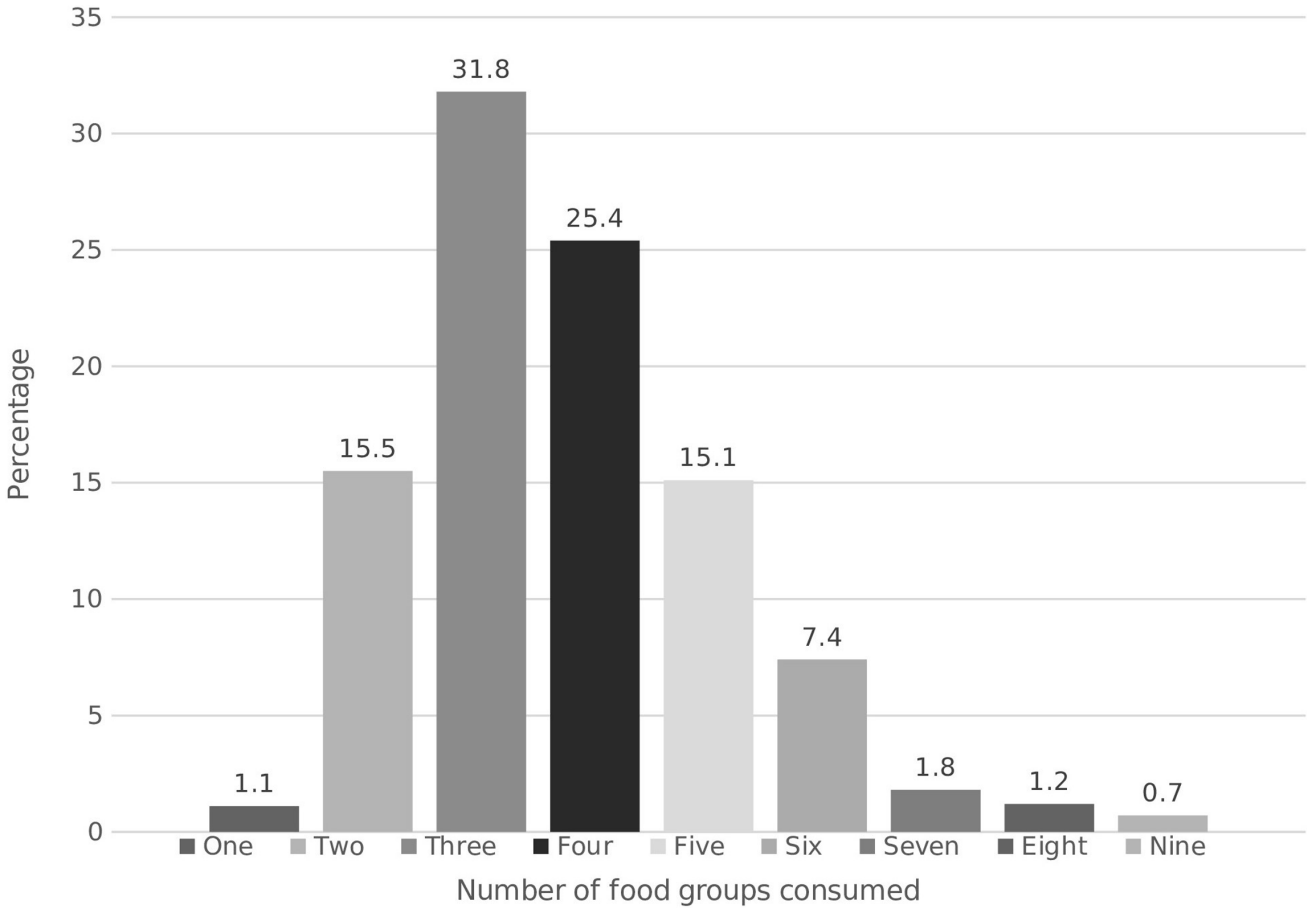
Dietary Diversity of women in Kersa, Ethiopia 2019.

**Figure 2:**
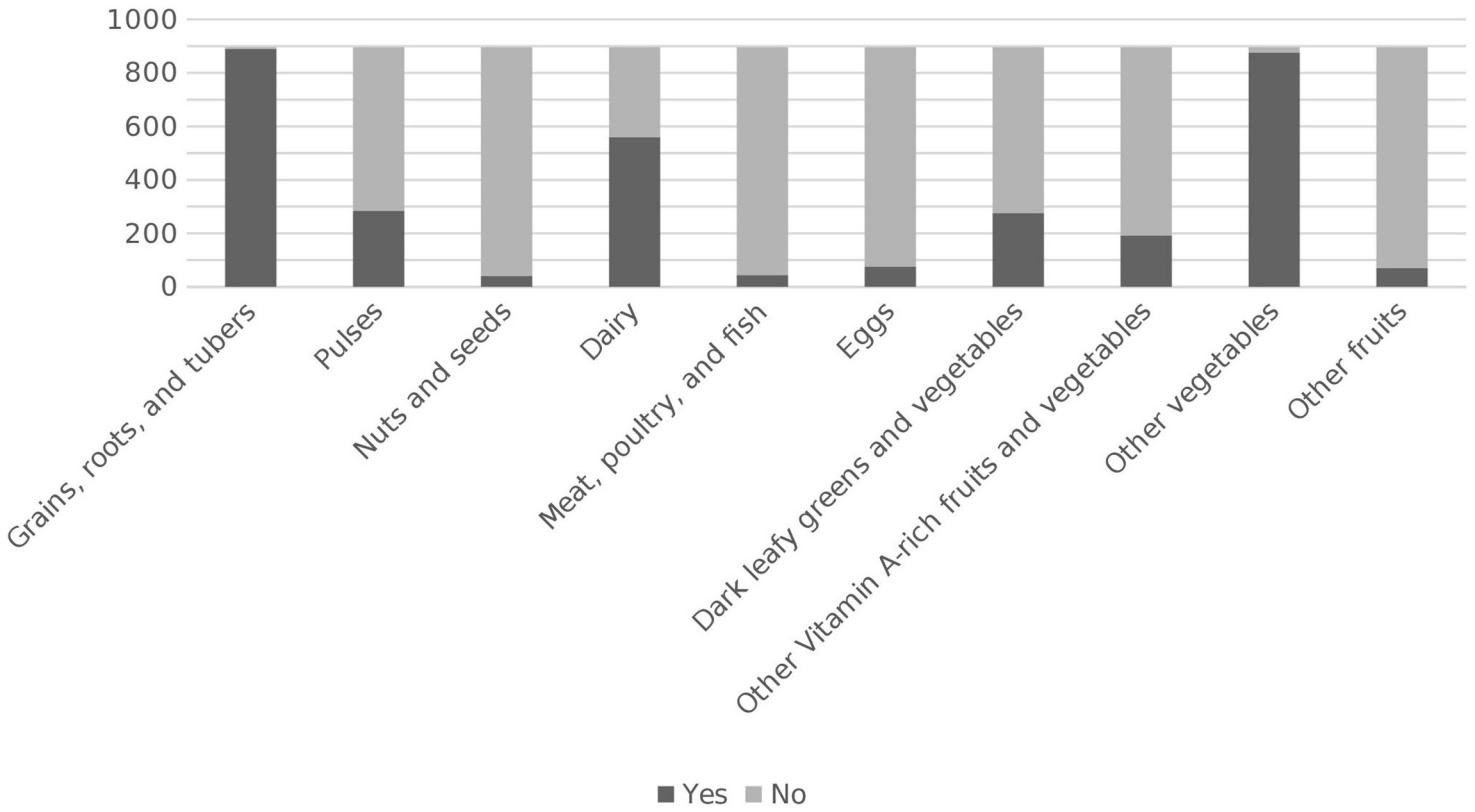
Consumption of food groups among women of reproductive age women in Kersa Ethiopia 2019.

Three out of four women in Kersa had a low dietary diversity (LDD), whereas only 220 (24.5%) achieved the recommended minimum dietary diversity of five or more food groups out of 10 per day. Median dietary diversity was low, with women consuming three out of 10 possible food groups Figure 1. Almost all participants reported consuming “grains, roots and tubers” and other vegetables. 559 (62.4%) participants consumed dairy products. The least frequently (less than 10%) consumed food groups were “meat, fish and poultry,” “nuts and seeds,” “eggs,” and other fruits Figure 2.

### Protein and energy intake

The median protein intake was 41.3 (32.9, 52.6) grams/day or 0.8 (0.6, 1.0) grams/ kilogram of body weight/day. Most dietary proteins were plant-based, with a median of 32.9 (26.1, 41.5) grams/day, while the median animal protein intake was 9.2 (2.0, 12.8) grams/day. Only 33.7% of reproductive-age women reported meeting the RDA for protein. Only 2.4% of total energy intake was derived from animal protein, while 10.1% was from plant protein. Most women, 695 (77.5%), consumed energy less than or equal to 1700 kcal /day Table 2.

**Table 2:** Intake of protein, other macro-nutrients, and total energy among women in Kersa, 2019.

Nutrients	Median intake grams/day (25th and 75th percentiles)	Median intake grams/ kilogram of body weight/day (25th and 75th percentiles)	Recommended dietary allowance (RDA)
Total Protein (grams)	41.3 (32.9, 52.6)	0.8 (0.6, 1.0)	46 gram/day or 0.8 grams/ kilogram of body weight/day. For lactating women from 1.1–1.3 gram/kilogram of body weight/day (37)
Animal-source protein (grams)	9.2 (2.0, 12.8)	0.2 (0.1, 0.3)	
Plant-based protein (grams)	32.9 (26.1, 41.5)	0.6 (0.5, 0.8)	
Carbohydrate (grams)	253.3 (201.3, 316.6)	4.9 (3.8, 6.1)	130 gram/day (37)
Fat (gram)	25.5 (18.9, 33.0)	0.5 (0.4, 0.7)	Data not available for RDA of fat (37)
Total Energy (Kcal)	1352.6 (1089.8, 1649.9)		1,900–2,200 calories per day (42). Energy requirements varies based on factors such as age, height, weight, physical activity level, lactation status, and overall health status

### Global dietary quality score (GDQS)

GDQS scores show 255 (28.4%) of participants are with a low risk of both nutrient adequacy and diet-related NCD risk, 630 (70.2%) were at moderate risk, and 12 (1.3%) were at high risk. The overall diet quality score ranges from 14 to 33; the median score was 20.5 (18.5, 23.0). The GDQS+ sub-metric score, which has 16 healthy food groups, ranges from 2 to 19 with a median of 6.5 (3.5, 9.3). On the other hand, the GDQS- sub metric, the 9 GDQS food groups of unhealthy or unhealthy in excessive amounts, has a range from 9 to 16. The median score of GDQS- sub-metric was 14 (13.0, 16.0).

### Association between dietary protein intake and overweight/obesity

Being overweight and obese was inversely associated with dietary protein intake in adjusted models. Reproductive age women who consumed a moderate amount of dietary protein had a significantly lower likelihood of being overweight or obese, with AOR of 0.21 (95% CI 0.10–0.48). Similarly, those who consumed a high amount of dietary protein had even lower odds, with AOR of 0.03 (95% CI 0.01–0.14), compared to those who consumed a low amount of dietary protein. On the other hand, the odds of being overweight and obese were three times (AOR = 3.33, 95% CI 1.24–8.95) higher among women in the age group of 40–49 years compared with women in 18–29 years. Similarly, non-farmers were three times (AOR = 3.24, 95% CI 1.54–6.82) more likely to be overweight and obese when compared to farmer women. Additionally, it was found that an increase in fat intake by one unit resulted in a 6% higher likelihood of being overweight or obese (AOR = 1.06, 95% CI 1.04–1.09). Furthermore, a unit increase in consumption of food groups from unhealthy or unhealthy in excessive amounts (GDQS-) has 30% (AOR = 1.30, 95% CI 1.05–1.61) increased likelihood of overweight and obesity. Finally, while the association was weak, there was a positive association between total carbohydrate intake and overweight and obesity (AOR = 1.005, 95% CI 1.00–1.01) Table 3.

**Table 3:** Association of protein intake with overweight and obesity while controlling for MDD-W, GDQS, sociodemographic, and other variables in Kersa, 2019.

Variables		Body mass index		COR with 95% CI	*p*-value	AOR with 95% CI	*p*-value
		Not overweight/obese	Overweight and Obese				
Protein intake (RDA)	Low	**405**	**41**	**Reference**		**Reference**	
	Moderate	**296**	**21**	**0.70 (0.41–1.21)**	**0.203**	**0.21 (0.10–0.48)**	**0.000**
	High	**129**	**5**	**0.38 (0.15–0.99)**	**0.047**	**0.03 (0.01–0.14)**	**0.000**
Occupation	Farmer	**697**	**37**	**Reference**		**Reference**	
	Non-farmer	**133**	**30**	**4.16 (2.47–7.03)**	**0.000**	**3.24 (1.54–6.82)**	**0.002**
Age	18–29	190	14	Reference		Reference	
	30–39	480	43	1.74 (0.93–3.25)	0.083	1.61 (0.81–3.19)	0.177
	40–49	160	14	3.46 (1.45–8.28)	0.005	3.33 (1.24–8.95)	0.017
MDD-W	Low	635	41	Reference		Reference	
	Adequate	194	26	2.08 (1.24–3.48)	0.006	1.13 (0.60–2.12)	0.71
Lactation status	Lactating	556	41	Reference		Reference	
	Non-lactating	274	26	1.39 (0.83–2.34)	0.208	1.26 (0.70–2.26)	0.438
Educational status	Informal	456	23	Reference		Reference	
	Formal	374	44	2.21 (1.31–3.75)	0.003	1.19 (0.60–2.38)	0.615
Wealth index	Poor	267	37	Reference		Reference	
	Middle	289	12	0.31 (0.16–0.60)	0.001	0.52 (0.29–1.17)	0.115
	Rich	274	18	0.50 (0.28–0.91)	0.023	0.94 (0.44–2.02)	0.878
Total fat intake (gram/day)				**1.03 (1.02–1.05)**	**0.000**	**1.06 (1.04–1.09)**	**0.000**
Total carbohydrate intake (gram/day)				**1.00 (0.99–1.01)**	**0.145**	**1.005 (1.00–1.01)**	**0.043**
GDQS+				0.98 (0.91–1.05)	0.558	1.06 (0.97–1.15)	0.203
GDQS-				**1.10 (0.93–1.30)**	**0.271**	**1.30 (1.05–1.61)**	**0.016**

Bold values: Variables with statistically significant association.

## Discussion

This study found that 7.5% of reproductive-age women in Kersa were overweight and obese. The recommended minimum dietary diversity of five or more food groups out of 10 per day was achieved by only 24.5% of women. Global Diet Quality Scores show 28, 70.8, and 1.2% of participants had a low, moderate, and high risk of nutrient adequacy and diet-related non-communicable diseases. Being overweight and obese was inversely associated with dietary protein intake per kilogram of body weight in adjusted models. Similarly, advancing age, non-farmers, higher consumption of food from unhealthy groups, and high fat intake were associated with overweight and obesity. Lastly, the study identified moderate dietary protein intake, non-farming occupation, middle age, better education, and wealth determinants of adequate diversity score.

The predominantly rural Kersa HDSS exhibited a higher prevalence of overweight and obesity (7.5%) among women of reproductive age compared to the national prevalence of 4% for rural residents in Ethiopia in 2016 (43). Notably, reports indicate a consistent rise in the prevalence of overweight and obesity in this age group, increasing from 3% in 2000 to 8% in 2016 in Ethiopia (44). The prevalence of overweight and obesity reached an alarming level in some developing countries such as 50.4% in Tanzania (45), 35.2% in Bangladesh (46), and 55.20% in Brazil (47). This shows the urgent need for targeted interventions and policies to address this growing issue and improve the health outcomes of women and their children.

In the current study, moderate and higher dietary protein intake is associated with low BMI among women of reproductive age. This is in line with similar studies that the consumption of high-protein diets produced more significant weight loss, lower BMI, loss of fat mass, and preserved lean mass compared with the consumption of normal-protein diets (48, 49). Unlike high fat and high carbohydrates (50), high protein intake, especially plant-based, does not result in excess energy and weight gain (51). Dietary protein is protective against overweight and obesity because it promotes satiety, energy expenditure and changes body composition in favor of fat-free body mass (52). However, the majority of reproductive age women in Kersa experience energy deficiency, which can be attributed to factors such as low socio-economic status, limited access to nutritious and diverse food options, and inadequate access to clean water and sanitation facilities. These factors can compromise the health and energy levels of women.

This study revealed that total carbohydrate intake has a weak but positive association with overweight and obesity among women of reproductive age. In this rural area, three fourth of energy was derived from carbohydrates such as foods made from cereals. However, in the body of the literature, there are controversies in selecting carbohydrates for weight reduction and reducing the risk of NCDs (53). On the other hand, a combination of low carbohydrates and high protein is emerging as an intervention to reduce body weight (54). Consistent with a critical review, women who consumed higher protein diets were less likely to be overweight and obese (55). This is due to high protein diets causing an increase in thermogenesis, decrease in fat absorption, and increase in fecal fat excretion (dairy products) or altering the intake of other nutrients, including carbohydrates and fats (56).

According to this study, the median dietary protein consumption for women in Kersa was found to be 0.8 grams per kilogram of body weight per day. This is slightly lower than the World Health Organization’s recommendation of 0.83 grams per kilogram of body weight per day to meet the needs of 97.5% of a healthy adult population. The study also found that lactating mothers in Kersa had slightly lower average daily dietary protein and energy intake compared to non-lactating women. This is in contrast to the WHO’s recommendation that lactating women should consume an additional 19 grams of protein per day in the first 6 months postpartum and 12.5 grams of protein per day after 6 months (57). Additionally, breastfeeding women are advised to consume an extra 500 kilocalories per day to compensate for the energy cost of lactation, in addition to the recommended energy intake for non-pregnant women (58).

The GDQS and dietary diversity are two important factors that are often associated with overweight and obesity. In Kersa, a unit increase in the consumption of food groups categorized as unhealthy or unhealthy in excessive amounts (GDQS-), increased the likelihood of overweight and obesity in 30% but dietary diversity was not associated with overweight and obesity. This is compatible with other similar studies (59, 60). However, some studies shows that higher dietary diversity increases the odds of obesity and overweight (61–63). It is important to be cautious when promoting health messages to the community, as consuming energy-dense foods from a variety of groups can lead to weight gain and obesity. On the other hand, women with lower are more likely to be overweight or obese, while those with higher GDQS have a lower risk (20, 59). Therefore, it is important to strike a balance between a high GDQS and appropriate dietary diversity in order to maintain a healthy weight and prevent overweight and obesity.

This study has provided valuable insights into the dietary protein intake and body mass index of reproductive-age women in Kersa. However, it is important to acknowledge that there are limitations to this study. Like all cross-sectional designs, it does not allow for causal inferences to be made. Therefore, future longitudinal studies should be conducted to examine dietary protein intake, as well as other macronutrients, energy intake, diet quality, and the entire range of body mass index in a similar population. Additionally, it is crucial to consider other factors such as lifestyle and behaviors, as the complex etiology of obesity cannot be fully explained by dietary protein intake alone. Lastly, it is worth noting that the Ethiopian food composition table did not include all the food items consumed in Kersa, so the Tanzanian food composition table was also used for missing food items. This may result in some differences in nutrient content between the two countries.

## Conclusions and recommendations

The study indicated an inverse relationship between body mass index (BMI) and dietary protein intake. It also revealed that women who consumed foods from unhealthy or unhealthy when consumed in excessive amount group were more likely to be overweight or obese. By increasing dietary protein consumption, reproductive age women can minimize the odds of becoming obese or overweight. Furthermore, it is crucial to implement interventions that encompass community-based educational programs that promote healthy eating, policy that support healthy food choices and increase accessibility of protein rich foods, and healthcare services to provide regular health screenings for women to identify underlying health concerns related to weight management and offer personalized guidance on incorporating dietary protein into their diets.

## Data availability statement

The original contributions presented in the study are included in the article/Supplementary material, further inquiries can be directed to the corresponding author.

## Ethics statement

The studies involving humans were approved by Institutional Health Research and Ethics Review Committee of College of Health and Medical Sciences, Haramaya University. The studies were conducted in accordance with the local legislation and institutional requirements. The participants provided their written informed consent to participate in this study.

## Author contributions

AR: Conceptualization, Data curation, Formal analysis, Investigation, Methodology, Project administration, Resources, Software, Supervision, Validation, Visualization, Writing – original draft, Writing – review & editing. NA: Conceptualization, Data curation, Funding acquisition, Investigation, Methodology, Project administration, Resources, Software, Supervision, Validation, Visualization, Writing – review & editing. KR: Conceptualization, Data curation, Investigation, Methodology, Software, Supervision, Validation, Visualization, Writing – review & editing. YD: Conceptualization, Data curation, Investigation, Software, Supervision, Validation, Writing – review & editing. EH: Conceptualization, Data curation, Investigation, Methodology, Software, Validation, Visualization, Writing – review & editing. WF: Conceptualization, Data curation, Funding acquisition, Investigation, Methodology, Software, Supervision, Validation, Visualization, Writing – review & editing.

## References

[1] Miller V Webb P Micha R Mozaffarian D. Defining diet quality: a synthesis of dietary quality metrics and their validity for the double burden of malnutrition. Lancet Planet Health. (2020) 4:e352–70. doi: 10.1016/S2542-5196(20)30162-5, PMID: 32800153 PMC7435701

[2] Gakidou E Afshin A Abajobir AA Abate KH Abbafati C Abbas KM. Global, regional, and national comparative risk assessment of 84 behavioural, environmental and occupational, and metabolic risks or clusters of risks, 1990–2016: a systematic analysis for the global burden of disease study 2016. Lancet. (2017) 390:1345–422. doi: 10.1016/S0140-6736(17)32366-8, PMID: 28919119 PMC5614451

[3] Health HTcSoP (2023). The best diet: quality counts. Available at: https://www.hsph.harvard.edu/nutritionsource/healthy-weight/best-diet-quality-counts/

[4] CDC (2023). Poor nutrition: National Center for Chronic Disease Prevention and Health Promotion (NCCDPHP). Available at: https://www.cdc.gov/chronicdisease/resources/publications/factsheets/nutrition.htm#:~:text=Consuming%20unhealthy%20food%20and%20beverages,in%20postmenopausal%20women%2C%20and%20colorectal 12731122

[5] Singh RB Isaza A Fatima G Maheshwari A Verma N Joshi S. The twelve fundamental dimensions of a high quality indo-Mediterranean diet. Scr Med. (2023) 54:1–7. doi: 10.5937/scriptamed54-42288

[6] Singh RB Takahashi T Fatima G Horiuchi R Fedacko J Huzova Z. Effects of antioxidant-rich indo-mediterranean foods on pre-heart failure: results from the Meta-analysis of randomized controlled trials. J Inflamm. (2020) 8:1–6. doi: 10.2174/1875041902008010001

[7] Bromage S Batis C Bhupathiraju SN Fawzi WW Fung TT Li Y. Development and validation of a novel food-based global diet quality score (GDQS). J Nutr. (2021) 151:75S–92S. doi: 10.1093/jn/nxab244 34689200 PMC8542096

[8] Fung TT Li Y Bromage S Bhupathiraju SN Batis C Fawzi W. Higher global diet quality score is associated with less 4-year weight gain in US women. J Nutr. (2021) 151:162S–7S. doi: 10.1093/jn/nxab170 34689192 PMC8542092

[9] Fallaize R Livingstone KM Celis-Morales C Macready AL San-Cristobal R Navas-Carretero S. Association between diet-quality scores, adiposity, total cholesterol and markers of nutritional status in European adults: findings from the Food4Me study. Nutrients. (2018) 10:49. doi: 10.3390/nu10010049, PMID: 29316612 PMC5793277

[10] George SM Ballard-Barbash R Manson JE Reedy J Shikany JM Subar AF. Comparing indices of diet quality with chronic disease mortality risk in postmenopausal women in the Women's Health Initiative observational study: evidence to inform national dietary guidance. Am J Epidemiol. (2014) 180:616–25. doi: 10.1093/aje/kwu173, PMID: 25035143 PMC4157698

[11] Ng M Fleming T Robinson M Thomson B Graetz N Margono C. Global, regional, and national prevalence of overweight and obesity in children and adults during 1980–2013: a systematic analysis for the global burden of disease study 2013. Lancet. (2014) 384:766–81. doi: 10.1016/S0140-6736(14)60460-8 24880830 PMC4624264

[12] Organization WH. World Health Organization factsheets, obesity and overweight, 2017. Geneva: WHO (2017).

[13] Amugsi DA Dimbuene ZT Mberu B Muthuri S Ezeh AC. Prevalence and time trends in overweight and obesity among urban women: an analysis of demographic and health surveys data from 24 African countries, 1991–2014. BMJ Open. (2017) 7:e017344. doi: 10.1136/bmjopen-2017-017344, PMID: 29079606 PMC5665233

[14] Dai H Alsalhe TA Chalghaf N Riccò M Bragazzi NL Wu J. The global burden of disease attributable to high body mass index in 195 countries and territories, 1990–2017: an analysis of the global burden of disease study. PLoS Med. (2020) 17:e1003198. doi: 10.1371/journal.pmed.1003198, PMID: 32722671 PMC7386577

[15] Knight JA. Diseases and disorders associated with excess body weight. Ann Clin Lab Sci. (2011) 41:107–21. PMID: 21844568

[16] Organization WH. Global health risks: Mortality and burden of disease attributable to selected major risks. Geneva: World Health Organization (2009).

[17] Mozaffarian D Hao T Rimm EB Willett WC Hu FB. Changes in diet and lifestyle and long-term weight gain in women and men. N Engl J Med. (2011) 364:2392–404. doi: 10.1056/NEJMoa1014296, PMID: 21696306 PMC3151731

[18] Moslehi N Ehsani B Mirmiran P Hojjat P Azizi F. Association of dietary proportions of macronutrients with visceral adiposity index: non-substitution and iso-energetic substitution models in a prospective study. Nutrients. (2015) 7:8859–70. doi: 10.3390/nu7105436, PMID: 26516906 PMC4632456

[19] Yeshaw Y Kebede SA Liyew AM Tesema GA Agegnehu CD Teshale AB. Determinants of overweight/obesity among reproductive age group women in Ethiopia: multilevel analysis of Ethiopian demographic and health survey. BMJ Open. (2020) 10:e034963. doi: 10.1136/bmjopen-2019-034963, PMID: 32156768 PMC7064084

[20] Bromage S Andersen CT Tadesse AW Passarelli S Hemler EC Fekadu H. The global diet quality score is associated with higher nutrient adequacy, midupper arm circumference, venous hemoglobin, and serum folate among urban and rural Ethiopian adults. J Nutr. (2021) 151:130S–42S. doi: 10.1093/jn/nxab264 34689198 PMC8564694

[21] Kalam F Gabel K Wiseman E Varady K. Alternate day fasting combined with a high protein/low carbohydrate diet: effect on body weight and metabolic disease risk factors in obese adults (P21-018-19). Current developments. Nutrition. (2019) 3:nzz041. doi: 10.1093/cdn/nzz041.P21-018-19

[22] Frestedt JL Zenk JL Kuskowski MA Ward LS Bastian ED. A whey-protein supplement increases fat loss and spares lean muscle in obese subjects: a randomized human clinical study. Nutr Metab. (2008) 5:1–7. doi: 10.1186/1743-7075-5-8 PMC2289832 18371214

[23] Richter CK Skulas-Ray AC Champagne CM Kris-Etherton PM. Plant protein and animal proteins: do they differentially affect cardiovascular disease risk? Adv Nutr. (2015) 6:712–28. doi: 10.3945/an.115.009654, PMID: 26567196 PMC4642426

[24] De Vries-Ten HJ Owolabi A Steijns J Kudla U Melse-Boonstra A. Protein intake adequacy among Nigerian infants, children, adolescents and women and protein quality of commonly consumed foods. Nutr Res Rev. (2020) 33:102–20. doi: 10.1017/S0954422419000222, PMID: 31997732 PMC7282859

[25] Aubertin-Leheudre M Adlercreutz H. Relationship between animal protein intake and muscle mass index in healthy women. Br J Nutr. (2009) 102:1803–10. doi: 10.1017/S0007114509991310, PMID: 19678968

[26] Neumann C Harris DM Rogers LM. Contribution of animal source foods in improving diet quality and function in children in the developing world. Nutr Res. (2002) 22:193–220. doi: 10.1016/S0271-5317(01)00374-8

[27] Shang X Scott D Hodge A English DR Giles GG Ebeling PR. Dietary protein from different food sources, incident metabolic syndrome and changes in its components: an 11-year longitudinal study in healthy community-dwelling adults. Clin Nutr. (2017) 36:1540–8. doi: 10.1016/j.clnu.2016.09.024, PMID: 27746001

[28] Iwamoto M Yagi K Yazumi K Komine A Shirouchi B Sato M. Eating a healthy lunch improves serum alanine aminotransferase activity. Lipids Health Dis. (2013) 12:1–7. doi: 10.1186/1476-511X-12-134 24034595 PMC3848840

[29] Hoffmann K Boeing H Dufour A Volatier J Telman J Virtanen M. Estimating the distribution of usual dietary intake by short-term measurements. Eur J Clin Nutr. (2002) 56:S53–62. doi: 10.1038/sj.ejcn.1601429, PMID: 12082518

[30] Assefa N Oljira L Baraki N Demena M Zelalem D Ashenafi W. HDSS profile: the Kersa health and demographic surveillance system. Int J Epidemiol. (2016) 45:94–101. doi: 10.1093/ije/dyv284, PMID: 26510420 PMC4795560

[31] Beasley JM Deierlein A Morland K Granieri E Spark A. Is meeting the recommended dietary allowance (RDA) for protein related to body composition among older adults?: results from the cardiovascular health of seniors and built environment study. J Nutr Health Aging. (2016) 20:790–6. doi: 10.1007/s12603-015-0707-5, PMID: 27709227 PMC5348248

[32] Assefa N Abdullahi YY Abraham A Hemler EC Madzorera I Dessie Y. Consumption of dietary folate estimates and its implication for reproductive outcome among women of reproductive age in Kersa: cross-sectional survey. BMC Nutr. (2021) 7:69. doi: 10.1186/s40795-021-00476-6, PMID: 34776012 PMC8591879

[33] Faul F Erdfelder E Lang A-G Buchner A. G* power 3: a flexible statistical power analysis program for the social, behavioral, and biomedical sciences. Behav Res Methods. (2007) 39:175–91. doi: 10.3758/BF03193146, PMID: 17695343

[34] Agren G Gibson RS. Food composition table for use in Ethiopia. Bokhandel: Almövist & Wiksell (1969).

[35] Lukmanji Z Hertzmark E Mlingi N Assey V Ndossi G Fawzi W. Tanzania food composition tables. Dar Es Salaam: MUHAS-TFNC (2008).

[36] Allowances RD. Recommended dietary allowances. Washington, DC: National Research Council-National Academy Press (1989).

[37] Institute of Medicine, Food and Nutrition Board, Standing Committee on the Scientific Evaluation of Dietary Reference Intakes, Subcommittee on Interpretation and Uses of Dietary Reference Intakes, Subcommittee on Upper Reference Levels of Nutrients, Panel on the Definition of Dietary Fiber. Dietary reference intakes for energy, carbohydrate, Fiber, fat, fatty acids, cholesterol, protein, and amino acids (macronutrients): National Academy of Sciences. J Am Diet Assoc. (2005) 102:1621–30. doi: 10.1016/s0002-8223(02)90346-9 12449285

[38] Jun S Cowan AE Dwyer JT Campbell WW Thalacker-Mercer AE Gahche JJ. Dietary protein intake is positively associated with appendicular lean mass and handgrip strength among middle-aged US adults. J Nutr. (2021) 151:3755–63. doi: 10.1093/jn/nxab288, PMID: 34494110 PMC8826630

[39] Project I. Data4Diets: Building blocks for diet-related food security analysis. Arlington, VA: USAID Advancing Nutrition (2018).

[40] Meyers LD Hellwig JP Otten JJ. Dietary reference intakes: The essential guide to nutrient requirements. Washington, DC: National Academies Press (2006).

[41] StataCorp L. Stata statistical software: Release, vol. 16. College Station, TX: StataCorp (2019).

[42] National Research Council, Commission on Life Sciences, Food and Nutrition Board. Subcommittee on the tenth edition of the recommended dietary Allowances. Recommended DIETARY Allowances: 10th edition (DIETARY REFERENCE INTAKES). 10th ed. Washington, DC: National Academies Press (1989).

[43] Mengesha Kassie A Beletew Abate B Wudu KM. Education and prevalence of overweight and obesity among reproductive age group women in Ethiopia: analysis of the 2016 Ethiopian demographic and health survey data. BMC Public Health. (2020) 20:1–11. doi: 10.1186/s12889-020-08941-w 32736617 PMC7393704

[44] Central Statistical Agency (CSA) [Ethiopia], ICF. Ethiopia Demographic and Health Survey. Addis Ababa, Ethiopia, and Rockville, Maryland: CSA and ICF (2016).

[45] Mosha D Paulo HA Mwanyika-Sando M Mboya IB Madzorera I Leyna GH. Risk factors for overweight and obesity among women of reproductive age in Dar Es Salaam, Tanzania. BMC Nutr. (2021) 7:1–10. doi: 10.1186/s40795-021-00445-z 34266482 PMC8283918

[46] Ahammed B Sarder MA Kundu S Keramat SA Alam K. Multilevel exploration of individual-and community-level factors contributing to overweight and obesity among reproductive-aged women: a pooled analysis of Bangladesh demographic and health survey, 2004–2018. Public Health Nutr. (2022) 25:2074–83. doi: 10.1017/S1368980022001124, PMID: 35570669 PMC9991804

[47] Lyrio AO Souza ES Conceição SDS Batista JE Brito SM Gomes Filho IS. Prevalence of overweight and obesity and associated factors among women of childbearing age in Brazil. Public Health Nutr. (2021) 24:5481–90. doi: 10.1017/S1368980021000409, PMID: 33500016 PMC10195318

[48] Hemler EC Bromage S Tadesse AW Zack R Berhane Y Canavan CR. Associations of percentage energy intake from total, animal and plant protein with overweight/obesity and underweight among adults in Addis Ababa. Ethiopia Public Health Nutr. (2022) 25:3107–20. doi: 10.1017/S1368980022001100, PMID: 35570670 PMC9991810

[49] Westerterp-Plantenga MS Lemmens SG Westerterp KR. Dietary protein - its role in satiety, energetics, weight loss and health. Br J Nutr. (2012) 108:S105–12. doi: 10.1017/S0007114512002589, PMID: 23107521

[50] Austin GL Ogden LG Hill JO. Trends in carbohydrate, fat, and protein intakes and association with energy intake in normal-weight, overweight, and obese individuals: 1971-2006. Am J Clin Nutr. (2011) 93:836–43. doi: 10.3945/ajcn.110.000141, PMID: 21310830

[51] Adams SH. Emerging perspectives on essential amino acid metabolism in obesity and the insulin-resistant state. Adv Nutr. (2011) 2:445–56. doi: 10.3945/an.111.000737, PMID: 22332087 PMC3226382

[52] Drummen M Tischmann L Gatta-Cherifi B Adam T Westerterp-Plantenga M. Dietary protein and energy balance in relation to obesity and co-morbidities. Front Endocrinol (Lausanne). (2018) 9:443. doi: 10.3389/fendo.2018.00443, PMID: 30127768 PMC6087750

[53] Naude CE Brand A Schoonees A Nguyen KA Chaplin M Volmink J. Low-carbohydrate versus balanced-carbohydrate diets for reducing weight and cardiovascular risk. Cochrane Database Syst Rev. (2022) 2022:CD013334. doi: 10.1002/14651858.CD013334.pub2 PMC8795871 35088407

[54] Hession M Rolland C Kulkarni U Wise A Broom J. Systematic review of randomized controlled trials of low-carbohydrate vs. low-fat/low-calorie diets in the management of obesity and its comorbidities. Obes Rev. (2009) 10:36–50. doi: 10.1111/j.1467-789X.2008.00518.x, PMID: 18700873

[55] Halton TL Hu FB. The effects of high protein diets on thermogenesis, satiety and weight loss: a critical review. J Am Coll Nutr. (2004) 23:373–85. doi: 10.1080/07315724.2004.10719381, PMID: 15466943

[56] Cao YJ Wang HJ Zhang B Qi SF Mi YJ Pan XB. Associations of fat and carbohydrate intake with becoming overweight and obese: an 11-year longitudinal cohort study. Br J Nutr. (2020) 124:715–28. doi: 10.1017/S0007114520001579, PMID: 32378502

[57] WHO, FAO, UNU. Protein and amino acid requirements in human nutrition. World Health Organ Tech Rep Ser. (2007) 935:1–265. 18330140

[58] FAO/WHO/UNU. Report of a joint FAO/WHO/UNU expert consultation. Rome: FAO.

[59] Paulo HA Mosha D Mwanyika-Sando M Mboya IB Madzorera I Killewo J. Role of dietary quality and diversity on overweight and obesity among women of reproductive age in Tanzania. PLoS One. (2022) 17:e0266344. doi: 10.1371/journal.pone.0266344, PMID: 35390059 PMC9045397

[60] Salehi-Abargouei A Akbari F Bellissimo N Azadbakht L. Dietary diversity score and obesity: a systematic review and meta-analysis of observational studies. Eur J Clin Nutr. (2016) 70:1–9. doi: 10.1038/ejcn.2015.118, PMID: 26220567

[61] Jayawardena R Byrne NM Soares MJ Katulanda P Yadav B Hills AP. High dietary diversity is associated with obesity in Sri Lankan adults: an evaluation of three dietary scores. BMC Public Health. (2013) 13:314. doi: 10.1186/1471-2458-13-314, PMID: 23566236 PMC3626879

[62] Mohajeri M Hoojeghani S Pourfarzi F Ghahremanzadeh M Barzegar A. Association between dietary diversity and obesity in Ardebil adults: a case-control study. Nutr Food Sci. (2020) 50:555–67. doi: 10.1108/NFS-04-2019-0118

[63] Zhang Q Chen X Liu Z Varma DS Wan R Zhao S. Diet diversity and nutritional status among adults in Southwest China. PLoS One. (2017) 12:e0172406. doi: 10.1371/journal.pone.0172406, PMID: 28231308 PMC5322886

