# Supplementary material for: Association between dietary protein intake, diet quality and diversity, and obesity among women of reproductive age in Kersa, Ethiopia
Source: Front Public Health. 2023 Nov 14;11:1258515. doi: 10.3389/fpubh.2023.1258515 (PMC10682820; doi:10.3389/fpubh.2023.1258515)
Supplement: Supplementary file 1 [file Table_1.docx]

## Questionnaire for Survey of Food Sources and Choices in Kersa, Ethiopia

**RESPONDENT IDENTIFICATION**

Directions: Respondent identifiers should be filled out by the interviewer after interviewer has introduced themselves to the household and presented a short synopsis of the research project. If more than one woman of reproductive age is living and present at the household, interviewer should randomly select one woman of reproductive age (18-49 years old) to answer the questionnaire. The woman who is selected should be the primary caregiver of the index child selected. Interviewer must obtain informed consent from the woman participating in survey. If an adult male (18-49 years) is living and present at the household, informed consent must be obtained from him as well. Consent forms should be completed before beginning survey.

1. **Household ID:**

__________________________________

1. **Name of Interviewer:**

__________________________________

1. **Woreda (district):**

__________________________________

__________________________________

1. **Today’s Date:**

__ __/__ __/__ __ __ __ *(DD/MM/YYYY)*

1. **Is there a child between 6 months to 59 months of age living in this household?**
   1. Yes
   2. No 🡪 end survey

*If there is more than one child between the ages of 6 to 59 months, please randomly selected a child present at the household to serve as the index child.*

1. **Child date of birth?** __ __/ __ __/ __ __ (DD/MM/YY)
2. **Age of child?** _____ months
3. **Is there a woman of reproductive age (18-49 yrs) who is the child’s primary caregiver present at the household?**
   1. Yes
   2. No 🡪 end survey
4. **Woman date of birth?** __ __/ __ __/ __ __ (DD/MM/YY)
5. **Age of woman?** ____ years

**WOMAN OF REPRODUCTIVE AGE QUESTIONNAIRRE**

*The following questions will be filled out by a woman of reproductive age who has consented to participate in the study. Unless otherwise specified, interviewer should read the questions and answers aloud to the participant and fill in or check the appropriate answers in the space provided. Questions will be in bold and answers in plain text. Directions for the interviewer will be italicized (do not read italicized text to participant)*

## A. DEMOGRAPHIC INFORMATION

| **Number** | **Question** | **Response** | **Code** |
| --- | --- | --- | --- |
| A1 | **How long have you lived in Kersa?** | ___ years |  |
| A2 | **What is your religion?** | 1. Orthodox 2. Muslim 3. Protestant 4. Catholic 5. Does not practice religion 6. Other | *If 1-5, skip to A4.*  *If 6, proceed to A3. other* |
| A3 | **If A2 is “other”, specify** | *Free text* |  |
| A4 | **What is the highest level of school you completed?** *Select one* | 1. Grade 1-10   Specify: __________________   1. Never attended school……………………99 2. Not finished first grade…………………………0 3. Technical or vocational training (TVET)…20 4. Preparatory school (grade 11-12) ……. 21 5. College diploma………………………………. 22 6. University/ college degree or high …… 23 |  |
| A5 | **What is your relationship to the head of household?** *Select one* | 1. Respondent is the head of household 2. Spouse 3. Sister 4. Daughter 5. In-law 6. Other relative 7. Non-relative | *If 1-5 or 7, skip to A6*  *If 6, proceed to A5.other* |
| A6 | **How would you describe your employment?** *Select one* | 1. No income generating activities (formal/informal) *🡪 skip to question 11* 2. Professional or technical/managerial……..1 3. Clerical…………….…2 4. Sales and services…3 5. Skilled manual………4 6. Unskilled manual……5 7. Agriculture……….....6 8. Small scale trade…..7 9. Medium scale trade…8 10. Other |  |
| A7 | **How would you describe this employment?** *Select one* | 1. Full time 2. Part time 3. Seasonal (only works certain times of the year) 4. Does not know |  |
| A8 | **In a typical work day, how many hours do you engage in incoming generating work?** | ______ hours | *All responses should be less than 20 hours* |
| A9 | **In a typical work week, how many hours do you engage in incoming generating work?** | ______hours | *All responses should be less than 100 hours* |
| **Partner Status** | | | |
| A10. | **What is your current marital status? Are you:** Select one | 1. Married – 2. Living with a partner, but not married 3. Single (never married, not living with partner)* 4. Widowed* 5. Divorced* 6. Separated* | *If 5-7 or 99, skip to question ____* |
| A11. | **How long have you been living with your partner?** *If less than 1 write 0. If dk write 888.* | _____ years |  |
| A12. | **What is the highest level of school your partner attended?** *Select one* | 1. Grade 1-10   Specify: __________________   1. Never attended school……………………99 2. Not finished first grade…………………………0 3. Technical or vocational training (TVET)…20 4. Preparatory school (grade 11-12) ……. 21 5. College diploma………………………………. 22 6. University/ college degree or high …… 23 |  |
| A13 | **How would you describe your partner’s employment?** *Select one* | 1. No income generating activities (formal/informal) *🡪 skip to question 11* 2. Professional or technical/managerial……..1 3. Clerical…………….…2 4. Sales and services…3 5. Skilled manual………4 6. Unskilled manual……5 7. Agriculture……….....6 8. Small scale trade…..7 9. Medium scale trade…8 10. Other |  |
|  |  |  |  |
| **Household Characteristics** | | | |
| A14.a | **How many children under 2 years of age live in your household?** | *Numeric* |  |
| A14.b | **How many children 2-4 years old live in your household** | *Numeric* |  |
| A14.c | **How many children 5-14 years old live in your household** | *Numeric* |  |
| A14.d | **How many adults (15 years or older) including yourself live in your household** | *Numeric* |  |
| A15. | **What type of housing do you live in?** **Do you:** *Select one* | 1. Own your own house 2. Own an apartment/condominium complex 3. Rent a house with a separate compound 4. Rent a room in a shared compound 5. Rent a room in a shared apartment/condominium complex 6. Shared one room 7. Staying with relatives 8. Do not have a home 9. Other | *If 1-5 or 99, skip to A16*  *If 6, proceed to A15.other* |
| A15.other | **If A15 is other, specify** | *Free text* |  |
| A16. | **Does any member of the household own land?** | 1. No 2. Yes | *If 1, then proceed to A17*  *If 0, skip to A19* |
| A17 | **What is the approximate size of this land?** | Numeric |  |
| A18 | **What are the units for size of land?** | 1. Acres  2. Hectares  3. Square meters |  |
| A19 | **What type of roof does your home have?** *Select one* | 1. Natural (no roof, thatched,dried leaves, mud) 2. Rudimentary all (plastic, bamboo, wood planks) 3. Finished (corrugated sheet, concrete/cement, wood planks/shingles) 4. Other 5. Does not know |  |
| A20 | **What type of floor does your home have?** *Select one* | 1. Natural floor (earth, sand, dung) 2. Rudimentary floor (Wood, pall, plastic cover) 3. Finished floor parquet or polished (wood, vinyl, asphalt, ceramic, cement, plastic tiles)Tiles 4. Does not know |  |
| A21 | **What is the main source of drinking water for your household?** *Select one* | 1. Piped into dwelling....................................11 2. Piped into compound ...............................12 3. Piped outside compound...........................13 4. Borehole……. .........................................21 5. Dug well 6. Protected well in the compound................31 7. Protected well outside the compound.......32 8. Unprotected well in the compound............33 9. Unprotected well outside the compound......34 10. Water from spring 11. Protected spring .....................................41 12. Unprotected spring..........................42 13. Rainwater.............................................51 14. Tanker truck ………………………………..61 15. Cart with small tank …………………………..71 16. Surface water 17. River/Lake/Pond/Stream/Dam …………81 18. Bottled water..........................….. ...…....91   99. Does not know | *If 1-9, or 99 then skip to A22.*  *If 10, then proceed to A21.other* |
| A21.other | **If A21 is other, then specify** | *Free text* |  |
| A22 | **Do you or any other members of your household do anything to make your water safe to drink?** | 1. No 2. Yes | *If 0, skip to A24* |
| A23 | **What do you/other members of your household usually do to make water safer to drink?** *Select all that apply* | 1. Boil 2. Add Bleach/Chlorine 3. Strain through a cloth 4. Use water filter 5. Solar disinfection 6. Let it stand and settle 7. Other 8. Do not know | *If 1-6 or 99, then skip to A24*  *If 7, proceed to A23.other* |
| A23.other | **If A23 is other, then specify** | *Free text* |  |
| A24 | **What kind of toilet facility do members of your household usually use?** *If participant is not sure, ask to view toilet facilities and mark the appropriate answer. Select all that apply* | 1. Flush/pour to piped sewer system 2. Flush/pour to septic tank 3. Flush/pour to pit latrine 4. Flush to somewhere else 5. Flush, do not know where 6. Ventilated improved pit latrine 7. Pit latrine with slab 8. Pit latrine without slab/ open pit 9. Composite toilet 10. Bucket toilet 11. Hanging toilet/latrine 12. No facility/bush/field 13. Other (specify) _______________ | *If 1-11, then skip to A26*  *If 12, proceed to A25* |
| A24.other | **If A24 is other, then specify** | *Free text* |  |

**A25. Does your household have the following:** *Select one*

|  | **Yes** | **No** | **Do not know** |
| --- | --- | --- | --- |
| **A25.a.** Working electricity | 1 | 0 | 8 |
| **A25.b.** Working generator | 1 | 0 | 8 |
| **A25.c**. Running water | 1 | 0 | 8 |
| **A25.d.** Sofa or couch | 1 | 0 | 8 |
| **A25.e**. Television | 1 | 0 | 8 |
| **A25.f.** Radio | 1 | 0 | 8 |
| **A25.g.** Refrigerator | 1 | 0 | 8 |
| **A25.h.** Mitad | 1 | 0 | 8 |
| **A25.i.** Bicycle | 1 | 0 | 8 |
| **A25.j.** Car | 1 | 0 | 8 |
| **A25.k.** Telephone/Cell Phone | 1 | 0 | 8 |

## B. Woman Health

*[Enumerator]*: Now I would like to ask some questions about your health and pregnancies.

|  | **Question** | **Response** | **Code** |
| --- | --- | --- | --- |
| B1. | **Are you pregnant and/or lactating?** | 1. Pregnant 2. Lactating 3. Pregnant and lactating 4. Non-pregnant, non-lactating |  |
| B2. | **How many pregnancies have you ever had, including your current pregnancy if you are currently pregnant?** | ___ ___ pregnancies |  |
| B3. | **How many live born children have you ever had?** | ___ ___ children |  |
| B4.a | **Have you ever been diagnosed with cardiovascular disease?** | 1. No 2. Yes   99. Don’t Know |  |
| B4.b | **Have you ever been diagnosed with diabetes?** | 1. No 2. Yes   99. Don’t Know |  |
| B4.c | **Have you ever been diagnosed with hypertension?** | 1. No 2. Yes   99. Don’t Know |  |
| B4.d | **Have you ever been diagnosed with Tuberculosis?** | 1. No 2. Yes   99. Don’t Know |  |
| B5 | **Do you have any family members diagnosed with diabetes?** | 1. No 2. Yes   99. Don’t Know |  |
| B6 | **Do you have any family members diagnosed with hypertension?** | 1. No 2. Yes   99. Don’t Know |  |

## C. Women’s FFQ

C1. **What best characterizes yesterday?**

1. Typical day
2. Fasting day
3. Holiday
4. Special event such as wedding
5. Other (please specify_____________)

*Ask woman respondent questions C.2.a-C.2.c about each food listed below, beginning with “Teff”.*

|  | **FOOD** | C.2.a Did you consume [FOOD] in the last 7 days?  0. No 1. Yes  *If “0”🡪 next food* | C.2.b How many days in the past 7 days did you consume [FOOD]?  *____days* | C.2.c Did you consume [FOOD] yesterday?  0. No 1. Yes |
| --- | --- | --- | --- | --- |
| Foods made from grains (such as injera, bread, porridge) | Teff |  |  |  |
|  | Maize |  |  |  |
|  | Rice |  |  |  |
|  | Wheat |  |  |  |
|  | Sorghum |  |  |  |
|  | Barley |  |  |  |
|  | Amaranth grain |  |  |  |
|  | Pasta |  |  |  |
|  | Other foods made from grain (specify) |  |  |  |
| Roots and tubers and plantains | Plantains |  |  |  |
|  | Enset |  |  |  |
|  | White or yellow-sweet potato |  |  |  |
|  | Yam |  |  |  |
|  | White cassava |  |  |  |
|  | Other white starchy staples (specify) |  |  |  |
| Pulses (beans, peas and lentils) | Common beans |  |  |  |
|  | Chickpeas |  |  |  |
|  | Lentils |  |  |  |
|  | Peas |  |  |  |
|  | Cowpea |  |  |  |
|  | Bambara |  |  |  |
|  | Other beans, peas, or bean/pea products (specify) |  |  |  |
| Nuts and seeds | Sunflower seeds |  |  |  |
|  | Sesame seeds |  |  |  |
|  | Groundnuts |  |  |  |
|  | Other nuts or seeds (specify______) |  |  |  |
| Dark green leady vegetables | Kale |  |  |  |
|  | Spinach |  |  |  |
|  | Amaranth leaves |  |  |  |
|  | Cassava leaves |  |  |  |
|  | Sweet potato leaves |  |  |  |
|  | Other dark leafy greens, incl. foraged leaves (specify) |  |  |  |
| Vitamin A-rich vegetables, roots and tubers | Pumpkin |  |  |  |
|  | Carrots |  |  |  |
|  | Squash |  |  |  |
|  | Orange-fleshed sweet potato |  |  |  |
|  | Yellow cassava (improved variety) |  |  |  |
|  | Other vitamin A rich vegetables that are orange or red (e.g. red sweet pepper) (specify) |  |  |  |
| Vitamin-A rich fruits | Ripe mango |  |  |  |
|  | Guava |  |  |  |
|  | Ripe papaya |  |  |  |
|  | Cantaloupe |  |  |  |
|  | Other locally available orange fruits (specify) |  |  |  |
|  | 100% fruit juice made from these orange fruits |  |  |  |
| Other vegetables | Tomato |  |  |  |
|  | Onion |  |  |  |
|  | Eggplant |  |  |  |
|  | Green pepper |  |  |  |
|  | Cucumber |  |  |  |
|  | Cabbage |  |  |  |
|  | Other local vegetables (specify) |  |  |  |
| Other fruits | Ripe banana |  |  |  |
|  | Pineapple |  |  |  |
|  | Apple |  |  |  |
|  | Orange |  |  |  |
|  | Other local and/or wild fruits (specify) |  |  |  |
|  | 100% fruit juice made from these other fruits |  |  |  |
| Red palm oil | Red palm oil |  |  |  |
| Milk and milk products | Milk |  |  |  |
|  | Cheese |  |  |  |
|  | Yogurt |  |  |  |
|  | Other milk products, NOT incl. ice cream and butter (specify) |  |  |  |
| Organ meat | Liver |  |  |  |
|  | Other organ meat (kidney, heart, intestines) (specify) |  |  |  |
| Meat and poultry | Beef meat |  |  |  |
|  | Goat meat |  |  |  |
|  | Chicken meat |  |  |  |
|  | Other poultry meat (specify) |  |  |  |
|  | Lamb |  |  |  |
|  | Other flesh meat (specify) |  |  |  |
| Fish and seafood | Fish or dried fish, shellfish or seafood |  |  |  |
| Eggs | Chicken eggs |  |  |  |
|  | Other eggs (specify) |  |  |  |
| Other oils and fats | Butter |  |  |  |
|  | Vegetable oil (fortified) |  |  |  |
|  | Vegetable oil (unfortified) |  |  |  |
|  | Other oil/fats for cooking and adding to food (specify) |  |  |  |
| Savory and fried snacks | Crisps, chips, French fries, fried dough and other fried foods |  |  |  |
| Sweets and sweetened beverages | Vitamin A-fortified sugar |  |  |  |
|  | Unfortified Sugar/honey (including use in beverages) |  |  |  |
|  | Cakes/Candies/chocolates/sweet biscuits |  |  |  |
|  | Other sweets (specify________) |  |  |  |
|  | Sweetened juice, soda and other sugar-sweetened beverages |  |  |  |
| Condiments and Seasonings | Iodized salt *(if they do not know, can check container if available)* |  |  |  |
|  | Non-iodized salt |  |  |  |
|  | Hot sauce, fish sauce, chilies, spices, herbs, tomato paste, flavor cubes, or other condiments |  |  |  |
| Other beverages and foods | Tea |  |  |  |
|  | Coffee |  |  |  |
|  | Clear broth |  |  |  |
|  | Alcohol |  |  |  |
|  | Any other foods (specify) |  |  |  |

## D. FOOD CHOICES AND COOKING PRACTICES

|  | **Question** | **Response** | **Code** |
| --- | --- | --- | --- |
| D1 | **Who in the household is primarily responsible for food procurement?** *Select all* | - 1. Participant   2. Head of household   3. Sibling   4. Offspring   5. Mother   6. Grandmother   7. Other relative   8. Other nonrelative |  |
| D2 | **What is the primary source of food for your household?** *Select one* | 1. Household production 2. Street vendor 3. Local Market 4. Grocery or supermarket 5. Other   99. Does not know | *If 1-3, skip to question D3*  *If 4, proceed to D2.other*  *If 99, skip to question D4* |
| D2.other | **If D2 is other, specify** | *Free text* |  |
| D3. | **How far is your primary food source from your house?** *If does not know, ask participant to estimate distance. If still does not know write 8888.* | _________ meters |  |
| D4. | **What is the secondary source of food for your household?** *Select one* | 1. No secondary source of food 2. Household production 3. Street vendor 4. Local Market 5. Grocery or supermarket 6. Other 7. Does not know | *If 1 or 6, skip to D6*  *If 2-4, skip to D5*  *If 5, proceed to D4.other* |
| D4.other | **If D4 is other, specify** | *Free text* |  |
| D5. | **How far is your secondary food source from your house?** *If does not know, ask participant to estimate distance. If still does not know write 8888.* | _____ meters |  |
| D6. | **On average, how much time do you and/or another household member spend procuring food per week?** *Select one* | 1. Less than 1 hour 2. 1-3 hours 3. 4-6 hours 4. 7-10 hours 5. More than 10 hours   99. Do not know |  |
| D7 | **On average, how many people eat in your household every day?** | _____ people |  |
| D8 | **How much money does your household usually spend buying food for the household each week?** *If do not know, then write 888,888* | __ __ __, ___ ___ __ birrs |  |
| D9 | **Are there children in the household who attend school?** | 1. No 2. Yes | *If 0, skip to D11* |
| D10 | **Do these children receive lunch at school** | 1. No 2. Yes |  |
| D11 | **On average, how many days per week does your household consume meat (livestock, poultry, or fish) at home?** *Select one* | 1. Never 2. 1-2 3. 3-4 4. 5-6 5. Everyday 6. Does not know |  |
| D12 | **Are you responsible for cooking/meal preparation for the household?** | 1. No 2. Yes | *If 0, skip to D14* |
| D13 | **How many hours per day do you spend cooking?** | 1. Less than 1 hour 2. 1-2 hours 3. 3-4 hours 4. 4-5 hours 5. more than 5 hours | *Skip to question D15 after answering this question* |
| D14 | **If someone other than yourself, who else is responsible for cooking/meal preparation?** | 1. Head of household 2. Sibling 3. Offspring 4. Mother 5. Grandmother 6. Other relative 7. Other nonrelative |  |
| D15 | **Which of the following is used in your household as the main source of cooking fuel each day?** *Select one* | 1. Electric 2. Gas 3. Paraffin 4. Charcoal 5. Firewood 6. Crop residuals, straw, grass, animal dung 7. Other 8. Does not know |  |
| D16 | **Where is your cooking stove/fire?** *Select one* | - 1. In the main house   2. In a separate building   3. Outdoors sheltered   4. Outdoors unsheltered |  |
| D17 | **What type of fat is usually used for cooking at home?** *Select all* | 1. Vegetable oil 2. Butter 3. Ghee 4. Margarine 5. Olive oil 6. Palm oil 7. Vegetable shortening 8. Animal fat 9. Coconut oil 10. Soy oil 11. Sesame oil 12. Other | *If 1-9, Skip to D18*  *If 10, proceed to D17.other* |
| D17.other | **If D17 is other, specify** | *Free text* |  |
| D18 | **In the past month, how often do you eat fried food prepared at home**? *Select one* | 1. Never 2. Less than once a week 3. 1-3 times per week 4. 4-6 times per week 5. Daily |  |
| D19 | **What is the most common method of food preparation in your household?** *Select one* | 1. Frying 2. steaming 3. boiling 4. roasting 5. Other | *1-4, skip to D20*  *If 5, proceed to D19.other* |
| D19.other | **If D19 is other, specify** | *Free text* |  |
| D20 | **In the past month, how often do you consume food outside the home**? | 1. Never 2. Less than once a week 3. 1 time per week 4. 2-4 times per week 5. 5-6 times per week 6. Daily |  |
| D21 | **In the past month, how often do you consume street or fast food, such as Pasti, Sambusa, Chips?** *Select one* | 1. Never 2. Less than once a week 3. 1 time per week 4. 2-4 times per week 5. 5-6 times per week 6. Daily |  |
| D22 | **Have you seen or heard a print, television, or radio advertisement for food items in the past week?** | 1. No 2. Yes | *If No, Skip to D24* |
| D23 | **If yes, for which food categories did you see or hear advertisements?** *Select all that apply* | 1. Starches  2. Pulses (beans, peas and lentils)  3. Meat (fish, poultry, beef)  4. Fruit  5. Vegetable  6. Eggs  7. Nuts and Seeds  8. Dairy  9. Savory Snacks (crisps, chips, French fries)  10. Sweets (cake, biscuits, candies)  11. Sugary Beverages |  |

**D24. How important are each of the following factors when making household food choices.** *Select one option for each category*

|  | **Not important** | **Somewhat important** | **Important** | **Very Important** |
| --- | --- | --- | --- | --- |
| D24.a **Price** | 1 | 2 | 3 | 4 |
| D24.b **Convenience** | 1 | 2 | 3 | 4 |
| D24.c **Availability at Market** | 1 | 2 | 3 | 4 |
| D24.d **Household Preference (mood or taste)** | 1 | 2 | 3 | 4 |
| D24.e **Nutrition/Health** | 1 | 2 | 3 | 4 |
| D24.f **Produced at home** | 1 | 2 | 3 | 4 |
| D24.g **Marketing/Advertising** | 1 | 2 | 3 | 4 |

|  | **Question** | **Response** | **Code** |
| --- | --- | --- | --- |
| D25 | **Are there any other factors associated with making household food choices that are not listed above?** | 1. No 2. Yes | *If 0, skip to D27* |
| D26 | I**f yes, what are other factors associated with making household food choices?** | *Free Text* |  |

**D27. Please indicate your preference for each food group***. Select a number for each food group.*

| **Food Group** | **Dislike Extremely** | **Dislike** | **Neither like nor dislike** | **Like** | **Like Extremely** |
| --- | --- | --- | --- | --- | --- |
| D27.a. **Grains, white roots and tubers, and plantains** | 1 | 2 | 3 | 4 | 5 |
| D27.b. **Pulses** *(beans, peas and lentils)* | 1 | 2 | 3 | 4 | 5 |
| D27.c. **Nuts and seeds** | 1 | 2 | 3 | 4 | 5 |
| D27.d. **Meat** *(red meat, pork, lamb, beef)* | 1 | 2 | 3 | 4 | 5 |
| D27.e **Poultry** | 1 | 2 | 3 | 4 | 5 |
| D27.f. **Fish** | 1 | 2 | 3 | 4 | 5 |
| D27.g **Eggs** | 1 | 2 | 3 | 4 | 5 |
| D27.h **Fruit** | 1 | 2 | 3 | 4 | 5 |
| D27.i **Vegetable** | 1 | 2 | 3 | 4 | 5 |
| D27.j **Dairy** | 1 | 2 | 3 | 4 | 5 |
| D27.k **Savory and fried snacks**  *Hint: Crisps, chips, French fries, fried dough and other fried foods* | 1 | 2 | 3 | 4 | 5 |
| D27.l **Sweets**  *Hint: Cakes, Candies, Chocolates, sweet biscuits* | 1 | 2 | 3 | 4 | 5 |
| D27.m **Sugar Sweetened Beverages**  *Hint: Sweetened juice, soda, and other sugar-sweetened beverages* | 1 | 2 | 3 | 4 | 5 |

D28. **Are there any foods or beverages that you avoid eating?**

- 1. Yes
  2. No 🡪 *skip to section E*

D29. **If yes, please list foods that you avoid eating**

| **Food Item** | **Reason for avoiding.** *Select all that apply* |
| --- | --- |
|  | 1. Cultural 1. Religious 2. Health 3. Other, Specify _________________ |
|  | 1. Cultural 1. Religious 2. Health 3. Other, Specify _________________ |
|  | 1. Cultural 1. Religious 2. Health 3. Other, Specify _________________ |
|  | 1. Cultural 1. Religious 2. Health 3. Other, Specify _________________ |
|  | 1. Cultural 1. Religious 2. Health 3. Other, Specify _________________ |

## E. Household Food Insecurity

*Ask of each month below. Begin with the current month of the previous year.*

*E1. Record starting month:*

1. *Maskarram*
2. *Tekemt*
3. *Hadar*
4. *Tahsas*
5. *Tarr*
6. *Yekatit*
7. *Makawit*
8. *Miaziah*
9. *Genbot*
10. *Sanni*
11. *Hamle*
12. *Nashi*
13. *Quaggimi*

*Record starting year (Ethiopian calendar):* [ ][ ][ ][ ]

Y Y Y Y

E2. **Did you experience shortage of food in the [MONTH] the past year?**

| E.2.a | E2.b | E2.c | E2.d | E2.e | E2.f | E2.g | E2.f | E2.h | E2.i | E2.j | E2.k | E2.L |
| --- | --- | --- | --- | --- | --- | --- | --- | --- | --- | --- | --- | --- |
| **Maskarram** | **Tekemt** | **Hadar** | **Tahsas** | **Tarr** | **Yekatit** | **Makawit** | **Miaziah** | **Genbot** | **Sanni** | **Hamle** | **Nashi** | **Quaggimi** |
| 0. No  1. Yes | 0. No  1. Yes | 0. No  1. Yes | 0. No  1. Yes | 0. No  1. Yes | 0. No  1. Yes | 0. No  1. Yes | 0. No  1. Yes | 0. No  1. Yes | 0. No  1. Yes | 0. No  1. Yes | 0. No  1. Yes | 0. No  1. Yes |

| E3. **In the past four weeks, did you worry that your household would not have enough food?** *Please circle a number.* | Never……………………………………………………..  Rarely (once or twice in the past four weeks)……………  Sometimes (three to ten times in the past four weeks)…..  Often (more than ten times in the past four weeks)……... | 0  1  2  3 |
| --- | --- | --- |
| E4**.** **In the past four weeks, were you or any household member not able to eat the kinds of foods you preferred because of a lack of resources?** *Please circle a number.* | Never……………………………………………………..  Rarely (once or twice in the past four weeks)……………  Sometimes (three to ten times in the past four weeks)…..  Often (more than ten times in the past four weeks)……... | 0  1  2  3 |
| E5**. In the past four weeks, did you or any household member have to eat a limited variety of foods due to a lack of resources?** *Please circle a number.* | Never……………………………………………………..  Rarely (once or twice in the past four weeks)……………  Sometimes (three to ten times in the past four weeks)…..  Often (more than ten times in the past four weeks)……... | 0  1  2  3 |
| E6**. In the past four weeks, did you or any household member have to eat some foods that you really did not want to eat because of a lack of resources to obtain other types of food?** *Please circle a number.* | Never……………………………………………………..  Rarely (once or twice in the past four weeks)……………  Sometimes (three to ten times in the past four weeks)…..  Often (more than ten times in the past four weeks)……... | 0  1  2  3 |
| E7**.** **In the past four weeks, did you or any household member have to eat a smaller meal than you felt you needed because there was not enough food?** *Please circle a number.* | Never……………………………………………………..  Rarely (once or twice in the past four weeks)……………  Sometimes (three to ten times in the past four weeks)…..  Often (more than ten times in the past four weeks)……... | 0  1  2  3 |
| E8**. In the past four weeks, did you or any household member have to eat fewer meals in a day because there was not enough food?** *Please circle a number.* | Never……………………………………………………..  Rarely (once or twice in the past four weeks)……………  Sometimes (three to ten times in the past four weeks)…..  Often (more than ten times in the past four weeks)……... | 0  1  2  3 |
| E9**. In the past four weeks, was there ever no food to eat of any kind in your household because of lack of resources to get food?** *Please circle a number.* | Never……………………………………………………..  Rarely (once or twice in the past four weeks)……………  Sometimes (three to ten times in the past four weeks)…..  Often (more than ten times in the past four weeks)……... | 0  1  2  3 |
| E10**.** **In the past four weeks, did you or any household member go to sleep at night hungry because there was not enough food?** *Please circle a number.* | Never……………………………………………………..  Rarely (once or twice in the past four weeks)……………  Sometimes (three to ten times in the past four weeks)…..  Often (more than ten times in the past four weeks)……... | 0  1  2  3 |
| E11**.** **In the past four weeks, did you or any household member go a whole day and night without eating anything because there was not enough food?** *Please circle a number.* | Never……………………………………………………..  Rarely (once or twice in the past four weeks)……………  Sometimes (three to ten times in the past four weeks)…..  Often (more than ten times in the past four weeks)……... | 0  1  2  3 |

## F. Food Expenditures for Women

|  | **FOOD CATEGORY** | **A. Did the household consume any items from (*category*) in the last month?**  1. Yes  2. No  *If no, >>next food* | B. **What was the main source?**  1. Own production  2. Market  3. Hunting/  fishing/gathering  4. Received as gift  5. Received in exchange for labor  6. Food aid  7. Other (specify)  99. Don’t know | *C.* ***If “2” for B***  **What was the price the last time you purchased (*item*)?**  *Price in ETB per KG*  *If Dk, 88* | D. **What was the total household expenditure on (*category*) in the last month?**  *ETB*  *If DK, 88* |
| --- | --- | --- | --- | --- | --- |
| F1 | **Chicken** |  |  |  |  |
| F2 | **Other poultry** |  |  |  |  |
| F3 | **Beef** |  |  |  |  |
| F4 | **Lamb** |  |  |  |  |
| F5 | **Other meat** |  |  |  |  |
| F6 | **Fish or seafood** |  |  |  |  |
| F7 | **Chicken Eggs** |  |  |  |  |
| F8 | **Other Eggs** |  |  |  |  |
| F9 | **Pulses** (like beans, peas and lentils) |  |  |  |  |
| F10 | **Nuts and seeds** (like groundnuts, treenuts and sunflower seeds) |  |  |  |  |
| F11 | **Milk and milk products** (not butter) |  |  |  |  |
| F12 | **Fish and seafood** |  |  |  |  |
| F13 | **Red palm oil** |  |  |  |  |
| F14 | **Other oils and fats** (including butter) |  |  |  |  |

## G**. Infant and Young Child Health and Feeding**

|  | **Question** | **Response** | **Code** |
| --- | --- | --- | --- |
|  | **What is your relationship to [child name]?** | 1. Mother 2. Grandmother 3. Sibling 4. Aunt 5. Other Relative 6. Not related |  |
| G1 | **What is the Child’s sex?** | 0. Male  1. Female |  |
| G2 | **Has (*name*) had diarrhea in the last two weeks, that is, since (*day of the week*) of the week before last?** *Diarrhea is determined as perceived by mother or caregiver, or as three or more loose or watery stools per day, or blood in stool.* | 0. No  1. Yes  99. Don’t know | *If “0” or “2”, 🡪G3* |
| G2.a | **In the last two weeks (14 days), for how many days did *(name)* have diarrhea?** | ___ ___ days |  |
| G3 | **In the last 2 weeks, has the child had an illness with a cough at any time?** | 0. No  1. Yes  99. Don’t know | *If 0 or 2🡪G4* |
| G3.a | **In the last two weeks, for how many days did *(name)* have a cough?** | ___ ___ days |  |
| G4 | **In the last 2 weeks, has the child had a fever?** | 0. No  1. Yes  99. Don’t know | *If 0 or 2🡪G4* |
| G4.a | **In the last two weeks, for how many days did *(name)* have a fever?** | ___ ___ days |  |
| G5 | **In the last 2 weeks, has the child had vomiting?** | 0. No  1. Yes  99. Don’t know | *If 0 or 2🡪G6* |
| G5.a | **In the last two weeks, for how many days did *(name)* have vomiting?** | ___ ___ days |  |
| G6 | **Has *(child’s NAME)* ever been breastfed?** | 0. No  1. Yes  99. Don’t know | *If “0”, 🡪G8* |
| G7 | **At what age (in months) were solids or liquids besides breastmilk introduced into the child's diet, for example plain water, teas, gripe water, herbal medicines, or soft foods?***If less than 1 month, enter number of days. Otherwise, enter number of months and enter “0” for days.* | ___ ___ Days  ___ ___ Months  88. Only received Breastmilk  99. Don’t Know |  |
| G8 | **Did you attend any prenatal or antenatal care visits for your pregnancy with (*child name*), either at home or at a health center?** | 0. No  1. Yes  99. Don’t know | *If “0”, 🡪 Section H* |
| G8. a | **How many visits did you attend for [NAME]?** | Number of visits________ |  |

## H. Child Feeding

*Ask woman respondent questions A-C about index child’s consumption of each food listed below, beginning with “Teff”.*

|  | **FOOD** | A. Did [INDEX CHILD NAME] consume [FOOD] in the last 7 days?  0. No 1. Yes  *If “0”🡪next food* | B. How many days in the past 7 days did [INDEX CHILD NAME] consume [FOOD]?  *____days* | C. Did [INDEX CHILD NAME] consume [FOOD] yesterday?  0. No 1. Yes |
| --- | --- | --- | --- | --- |
| Foods made from grains (such as injera, bread, porridge) | Teff |  |  |  |
|  | Maize |  |  |  |
|  | Rice |  |  |  |
|  | Wheat |  |  |  |
|  | Sorghum |  |  |  |
|  | Barley |  |  |  |
|  | Amaranth grain |  |  |  |
|  | Pasta |  |  |  |
|  | Other foods made from grain (specify____________) |  |  |  |
| Roots and tubers and plantains | Plantains |  |  |  |
|  | Enset |  |  |  |
|  | White and yellow sweet potato |  |  |  |
|  | Yam |  |  |  |
|  | Cassava |  |  |  |
|  | Other white starchy staples (specify______) |  |  |  |
| Pulses (beans, peas and lentils) | Common beans |  |  |  |
|  | Chickpeas |  |  |  |
|  | Lentils |  |  |  |
|  | Peas |  |  |  |
|  | Cowpea |  |  |  |
|  | Bambara |  |  |  |
|  | Other beans, peas, or bean/pea products (specify______) |  |  |  |
| Nuts and seeds | Sunflower seeds |  |  |  |
|  | Sesame seeds |  |  |  |
|  | Groundnuts |  |  |  |
|  | Other nuts or seeds (specify______) |  |  |  |
| Dark green leafy vegetables | Kale |  |  |  |
|  | Spinach |  |  |  |
|  | Amaranth leaves |  |  |  |
|  | Cassava leaves |  |  |  |
|  | Sweet potato leaves |  |  |  |
|  | Other dark leafy greens, incl. foraged leaves (specify______) |  |  |  |
| Vitamin A-rich vegetables, roots and tubers | Pumpkin |  |  |  |
|  | Carrots |  |  |  |
|  | Squash |  |  |  |
|  | Orange-fleshed sweet potato |  |  |  |
|  | Yellow-fleshed cassava (improved variety) |  |  |  |
|  | Other vitamin A rich vegetables that are orange or red (e.g. red sweet pepper) (specify______) |  |  |  |
| Vitamin-A rich fruits | Ripe mango |  |  |  |
|  | Guava |  |  |  |
|  | Ripe papaya |  |  |  |
|  | Cantaloupe |  |  |  |
|  | Other locally available orange fruits (specify____________) |  |  |  |
|  | 100% fruit juice made from these orange fruits |  |  |  |
| Other vegetables | Tomato |  |  |  |
|  | Onion |  |  |  |
|  | Eggplant |  |  |  |
|  | Green pepper |  |  |  |
|  | Cucumber |  |  |  |
|  | Cabbage |  |  |  |
|  | Other local vegetables (specify_____) |  |  |  |
| Other fruits | Ripe banana |  |  |  |
|  | Pineapple |  |  |  |
|  | Apple |  |  |  |
|  | Orange |  |  |  |
|  | Other local and/or wild fruits (specify_______) |  |  |  |
|  | 100% fruit juice made from these other fruits |  |  |  |
| Red palm oil | Red palm oil |  |  |  |
| Milk and milk products | Milk |  |  |  |
|  | Cheese |  |  |  |
|  | Yogurt |  |  |  |
|  | Other milk products, NOT incl. ice cream and butter (specify_____) |  |  |  |
| Organ meat | Liver |  |  |  |
|  | Other organ meat (kidney, heart, intestines) (specify_____) |  |  |  |
| Meat and poultry | Beef meat |  |  |  |
|  | Goat meat |  |  |  |
|  | Chicken meat |  |  |  |
|  | Other poultry meat (specify______) |  |  |  |
|  | Lamb |  |  |  |
|  | Rabbit |  |  |  |
|  | Other flesh meat (specify______) |  |  |  |
| Fish and seafood | Fish or dried fish, shellfish or seafood |  |  |  |
| Eggs | Chicken eggs |  |  |  |
|  | Other eggs (specify______) |  |  |  |
| Other oils and fats | Butter |  |  |  |
|  | Vegetable oil (fortified) |  |  |  |
|  | Vegetable oil (unfortified) |  |  |  |
|  | Other oil/fats for cooking and adding to food (specify________) |  |  |  |
| Savory and fried snacks | Crisps, chips, French fries, fried dough and other fried foods |  |  |  |
| Sweets and sweetened beverages | Vitamin A-fortified sugar |  |  |  |
|  | Unfortified Sugar/honey (including use in beverages) |  |  |  |
|  | Cakes/Candies/chocolates/sweet biscuits |  |  |  |
|  | Other sweets (specify________) |  |  |  |
|  | Sweetened juice, soda and other sugar-sweetened beverages |  |  |  |
| Condiments and Seasonings | Iodized salt *(if they do not know, can check container if available)* |  |  |  |
|  | Non-iodized salt |  |  |  |
|  | Hot sauce, fish sauce, chilies, spices, herbs, tomato paste, flavor cubes, or other condiments |  |  |  |
| Other beverages and foods | Tea |  |  |  |
|  | Coffee |  |  |  |
|  | Clear broth |  |  |  |
|  | Alcohol |  |  |  |
|  | Any other foods (specify) |  |  |  |

## I.NUTRITIONAL KNOWLEDEGE: **

*Directions: Unless otherwise directed interviewer should read the participant the question and wait for the participant to respond to the question in their own words. Below the question is a list of potential right answers, along with “other” and “Does not know”. The surveyor should record the participants answer and then categorize it according to the predefined response options. Check all responses that apply.*

|  | **Question** | **Response** | **Code** |
| --- | --- | --- | --- |
| **I1** | **What is the first food a newborn baby should receive?** | 1. Only breastmilk 2. Other 3. Does not know |  |
| **I2** | **Have you heard about exclusive breastfeeding?** | 1. No 2. Yes | *If 0, skip to question I4* |
| **I3** | **How long is it recommended that a woman exclusively breastfeeds her child?** | 1. Less than six months 2. Six months (correct) 3. More than six months 4. Other 5. Does not know |  |
| **I4** | **What is the recommended number of food groups an adult should consume daily?** *Read participant potential answers and record their response.* | 1. 1-2 2. 3-4 3. 5 4. more than 5 |  |
| **I5** | **What are the signs of undernutrition?** | 1. Lack of energy/weakness: cannot work, study or play as normal (disability) 2. Weakness of the immune system (becomes ill easily or becomes seriously ill) 3. Loss of weight/thinness 4. Children do not grow as they should (growth faltering) 5. Other 6. Does not know |  |
| **I6** | **What are the health consequences of being overweight/obese*?*** | 1. Diabetes 2. High blood pressure 3. Cancer 4. Heart problems 5. Other 6. Does not know |  |
| **I7** | **What foods are sources of Protein?** | 1. Nuts and Seeds 2. Beans 3. Sorghum 4. Amaranth grain 5. Teff 6. Rice 7. Barley 8. Millet 9. Lentils 10. Chickpeas 11. Cowpea 12. Red kidney bean 13. Bambara 14. Mung beans 15. Sunflower seed 16. Sesame seed 17. Groundnuts 18. Milk 19. Cheese 20. Yogurt 21. Other 22. Does not know 23. Fish 24. Poultry 25. Beef 26. Meat 27. Eggs |  |

## J. HOMESTEAD FOOD PRODUCTION

**J1. Does your household have access to a plot of land where you could grow food?**

- 1. Yes
  2. No 🡪 *skip to question J3*

**J2. If yes, what is the approximate size of this plot of land?**

_________. __

**Selected units of area**

- - 1. Acres
    2. Square meters
    3. Hectares

**J3. Does your household participate in any agricultural activities** (cash crops, home gardening, livestock, or other)**?**

- 1. Yes
  2. No 🡪 Skip to Next section

**J4. Does your household participate in homestead food production** (food produced mainly for household consumption)**?**

1. Yes
2. No

**J5. Does your household currently own any livestock, herds, other farm animals, or poultry?**

- 1. Yes
  2. No 🡪 *skip to next section*
  3. Do not know

**J6. If yes, how many of the following animals does this household currently have?** *Read the categories to the participant and write down a number next to each category. If the household does not own any animals in a category write “0.” If the participant does not know write “88”.*

J17.a **Cattle, milk cows, or bulls** _______________

J17.b **Horses, donkeys, or mules** _______________

J17.c **Goats**  _______________

J17.d **Sheep**  _______________

J17.e **Chickens, ducks, hens** _______________

J17.f **Pigs** _______________

## K1: Women’s Anthropometry

*Take measurements for only the female respondent in the household. Make sure the women was sitting down before taking blood pressure. Wait 3 minutes in between blood pressure measurements. There are women who wear heavy clothes or a head cover and are unwilling to remove. If possible, ask woman to wear light clothing for measurement.*

| **NUM** | **Question** | **Result** |
| --- | --- | --- |
| **K.1.a1** | **Blood Pressure Measure 1** | \|__\|__\|__\|.\|__\| |
| **K.1.a2** | **Blood Pressure Measure 2** | \|__\|__\|__\|.\|__\| |
| K.1.b | **Clothing worn (0=none,1=very light,2=light, 3=med., 4=heavy) If not sure, specify:_________** | \|__\| |
| K.1.c | **Head cover, hair style, or other item worn on the head and unwilling to remove (Yes=1;No=0)** | \|__\| |
| K.1.d1 | **kg- Weight 1 (Measure to 1 decimal point)** | \|__\|__\|__\|.\|__\| |
| K.1.d2 | **kg- Weight 2 (Measure to 1 decimal point)** | \|__\|__\|__\|.\|__\| |
| K.1.e1 | **cm- Height 1 (Measure to 1 decimal point)** | \|__\|__\|__\|.\|__\| |
| K.1.e2 | **cm- Height 2 (Measure to 1 decimal point)** | \|__\|__\|__\|.\|__\| |
| K.1.f1 | **cm- MUAC 1 (Measure to 1 decimal point)** | \|__\|__\|.\|__\| |
| K.1.f2 | **cm- MUAC 2 (Measure to 1 decimal point)** | \|__\|__\|.\|__\| |
| K.1.g | **MUAC < 21cm for adult women?** | 0. No  1. Yes  **If “1”, refer** |
| K.1.e | **Referral given?** | 0. No  1. Yes |

## K2: Index Child Anthropometry

*ENUMERATOR: CONFIRM PERMISSION OF PARENT/CAREGIVER OF INDEX CHILD TO TAKE MEASUREMENTS. Build in a tablet check to make sure child is the index child; if not, list reason index child is unavailable NOTE: MEASURE A CHILD < 24 MONTHS LYING DOWN & MEASURE A CHILD > 24 MONTHS STANDING.* ***If child’s MUAC<11.5 cm,*** *refer to local health worker or to next EOS/ CHD/ or the local NGO or CHW.*

| **NUM** | **Question** | **Result** |
| --- | --- | --- |
| K.2.a | **Clothing worn (0=none,1=very light,2=light, 3=med., 4=heavy) If not sure, specify:_________** | \|__\| |
| K.2.b | **Head cover, hair style, or other item worn on the head and unwilling to remove (Yes=1;No=0)** | \|__\| |
| K.2.c1 | **kg- Weight 1 (Measure to 1 decimal point)** | \|__\|__\|__\|.\|__\| |
| K.2.c2 | **kg- Weight 2 (Measure to 1 decimal point)** | \|__\|__\|__\|.\|__\| |
| K.2.d1 | **cm- Height 1 (Measure to 1 decimal point)** | \|__\|__\|__\|.\|__\| |
| K.2.d2 | **cm- Height 2 (Measure to 1 decimal point)** | \|__\|__\|__\|.\|__\| |
| K.2.e1 | **cm- MUAC 1 (Measure to 1 decimal point)** | \|__\|__\|.\|__\| |
| K.2.e2 | **cm- MUAC 2 (Measure to 1 decimal point)** | \|__\|__\|.\|__\| |
| K.2.f | **MUAC < 11.5cm for children?** | 0. No  1. Yes  **If “1”, refer** |
| K.2.g | **Referral given?** | 0. No  1. Yes |
